# Superficial siderosis of the central nervous system: an usual cause of sensorineural hearing loss

**DOI:** 10.5935/1808-8694.20130044

**Published:** 2015-11-02

**Authors:** Diana Cunha Ribeiro, Joana Nunes, Ana Cláudia Ribeiro, Felisberto Maricato, Carlos Ribeiro

**Affiliations:** a MD. ENT Intern; b MD. Neuroradiology Intern; c MD. Neurology Intern; d MD. Assistant ENT physician; e Head of the Otorhinolaryngology Clinic. University and Hospital Center of Coimbra

**Keywords:** central nervous system, hearing loss, sensorineural, hemosiderosis, siderosis

## INTRODUCTION

The Superficial Siderosis (SS) of the Central Nervous System (CNS) is a rare clinical entity. It was first described in 1908, by Hamill, and there are up to 200 cases described so far^1^, ^2^.

It is secondary to chronic and recurrent hemorrhage in the subarachnoid space^1^, ^3^. The most affected areas are the cerebellum, the fron-tobasal lobe, the olfactory bulbs, the temporal cortex, the brainstem, cranial nerves, spinal cord and nerve roots. Free iron and hemosiderin deposits are highly toxic, causing neuronal lesion and reactive gliosis^2^.

There are two types of SS: idiopathic and secondary. The cause for hemorrhage is detected in almost half of the cases, and the most frequent causes are: highly vascularized cord tumors, CNS vascular malformations, surgery in the posterior fossa, head injury or prior intradural surgery^2^, ^3^, ^4^.

The classic clinical triad is bilateral sensorineural hearing loss, progressive cerebellum ataxia and pyramidal tract involvement^2^, ^3^, ^5^. Nonetheless, other symptoms may ensue: headaches, bladder disorders, dementia, anosmia, nystagmus, anterior horn syndrome and Parkinson's symptoms.

Age ranges between 14 and 77 years, becoming evident around 50 years in average, with the pre-clinical phase identification about 10 years before^1^, ^2^.

## CLINICAL CASE

A 54-year-old woman with clinical signs and symptoms of bilateral sensorineural hearing loss of unknown origin, with 15 years of evolution, followed by an ENT, with pure tone audiogram showing mild sensorineural hearing loss (SNHL) in the right ear and severe SNHL in her left ear, associated with changes in gait and balance disorders worsening for 10 years and, recently, memory loss.

Upon the objective exam, she had had disorders to her recent memory. The cranial nerves were normal, except for a reduction in hearing acuity and nystagmus in the extreme right gaze. Her motor exam showed bilateral ataxia and cerebellar dysarthria. She had live bone-tendon reflexes on her lower limbs, with Babinsky being positive in both sides.

**Figure 1:**
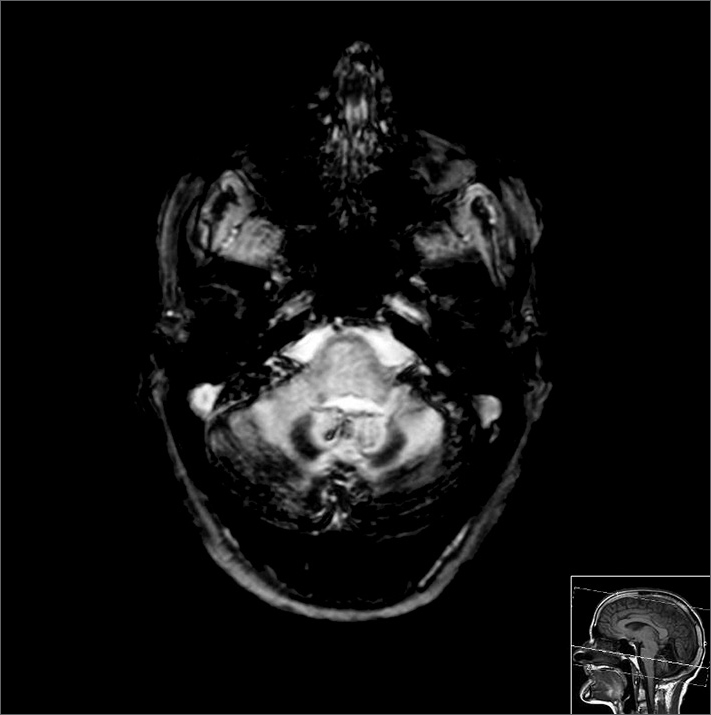
MRI - CE, axial slices in T2*, where we see intense hypo signal in T2* of the pial coating of all the structures in the posterior fossa, in the internal vertent of the occipital lobe and the Sylvian fissures, marked cerebellar atrophy, translating into superficial siderosis of the CNS.

We did the following diagnostic complementary tests: pure tone audiometry - which showed moderate sensorineural hearing loss on her right ear and profound SNHL on the left; brainstem auditory evoked potentials which were inconclusive because of numerous artifacts; analytical study with high ferritin and lumbar punction with xanthochromic CSF. MRI-CE (Figure 1) showed (hypo intensive signal in T2 and T2*, of the pial coating of all the posterior fossa structures, internal vertent of the occipital lobes and Sylvian fissures, and marked cerebellar atrophy, translating into CNS SS, spinal cord MRI showed “mild hypo signal in T2 involving the periphery of the entire cord” and the spinal cord angiography was normal.

Thus, we diagnosed idiopathic CNS SS, and the patient is being observed, having been medicated with vitamin E.

## DISCUSSION

When the ENT has a patient with progressive, bilateral sensorineural hearing loss, especially a young adult, the differential diagnosis must include various clinical entities, namely: neurosyphilis, multiple sclerosis, autoimmune causes and CNS SS^1^, ^2^.

The eight cranial nerve, is the one with the longest glial component, being particularly vulnerable to hemosiderin deposits^3^, ^5^.

The CSF is altered in about 75% of the cases, being xanthochromic or hemorrhagic, with high levels of protein iron and ferritin, and with siderophages^5^.

CNS MRI shows pathognomonic signs which may be detected in the pre-symptomatic phase of the disease.

Treatment, in cases of secondary SS, is geared towards the identifiable cause of hemorrhage. In idiopathic SS, treatment is symptomatic only, including Paracetamol and Piracetam^4^, ^5^ for the headaches and Selegilin and vitamin E in an attempt to reduce the oxidative toxic effect of the iron-heme complex^3^.

## FINAL REMARKS

The slow and dragging clinical course of the disease, its rarity and evolution without alarming signs, result in late diagnosis, at which point the CNS lesions are irreversible; therefore, a high level of suspicion is required.
